# Multi-Center Evaluation of Gel-Based and Dry Multipin EEG Caps

**DOI:** 10.3390/s22208079

**Published:** 2022-10-21

**Authors:** Chuen Rue Ng, Patrique Fiedler, Levin Kuhlmann, David Liley, Beatriz Vasconcelos, Carlos Fonseca, Gabriella Tamburro, Silvia Comani, Troby Ka-Yan Lui, Chun-Yu Tse, Indhika Fauzhan Warsito, Eko Supriyanto, Jens Haueisen

**Affiliations:** 1 Institute of Biomedical Engineering and Informatics, Technische Universität Ilmenau, 98693 Ilmenau, Germany; 2 Faculty of Information Technology, Monash University, Building 63, 25 Exhibition Walk, Clayton, VIC 3800, Australia; 3 Brain and Psychological Sciences Research Centre, Swinburne University of Technology, P.O. Box 218, Hawthorn, VIC 3122, Australia; 4 Faculdade de Engenharia, Universidade do Porto, Rua Dr. Roberto Frias, s/n, 4200-465 Porto, Portugal; 5 Institute of Science and Innovation in Mechanical and Industrial Engineering, LAETA/INEGI, 4200-465 Porto, Portugal; 6 BIND-Behavioral Imaging and Neural Dynamics Center, University “G. d’Annunzio” of Chieti–Pescara, Via Luigi Polacchi, 11, 66100 Chieti, Italy; 7 Department of Psychology, University of Lübeck, Ratzeburger Allee 160, 23562 Lübeck, Germany; 8 Center of Brain, Behavior and Metabolism, University of Lübeck, Marie-Curie-Straße, 23562 Lübeck, Germany; 9 Department of Social and Behavioural Sciences, City University of Hong Kong, Hong Kong, China; 10 IJN-UTM Cardiovascular Engineering Centre, School of Biomedical Engineering & Health Sciences, Universiti Teknologi Malaysia, Johor Bahru 81300, Malaysia

**Keywords:** electroencephalography, dry electrodes, multi-center, multi-operator, validation, evoked activity, resting state, brain imaging

## Abstract

Dry electrodes for electroencephalography (EEG) allow new fields of application, including telemedicine, mobile EEG, emergency EEG, and long-term repetitive measurements for research, neurofeedback, or brain–computer interfaces. Different dry electrode technologies have been proposed and validated in comparison to conventional gel-based electrodes. Most previous studies have been performed at a single center and by single operators. We conducted a multi-center and multi-operator study validating multipin dry electrodes to study the reproducibility and generalizability of their performance in different environments and for different operators. Moreover, we aimed to study the interrelation of operator experience, preparation time, and wearing comfort on the EEG signal quality. EEG acquisitions using dry and gel-based EEG caps were carried out in 6 different countries with 115 volunteers, recording electrode-skin impedances, resting state EEG and evoked activity. The dry cap showed average channel reliability of 81% but higher average impedances than the gel-based cap. However, the dry EEG caps required 62% less preparation time. No statistical differences were observed between the gel-based and dry EEG signal characteristics in all signal metrics. We conclude that the performance of the dry multipin electrodes is highly reproducible, whereas the primary influences on channel reliability and signal quality are operator skill and experience.

## 1. Introduction

Electroencephalography (EEG) is widely used in research and medical routine for the monitoring of brain activity by the acquisition of biopotentials on the human head surface. The advancement in the acquisition method from the gold standard gel-based electrode EEG caps to the more recent dry electrode EEG caps supported the considerable broadening of the field of applications, such as for clinical purposes [1,2], in brain–computer interfaces (BCI) [3,4,5,6,7], in mobile EEG [8,9,10,11], in neurofeedback applications [5,12,13], and for emergency situations [14,15]. The new fields of application have previously been considered challenging or impossible due to the inherent limitations of gel-based electrodes.

The need for gel electrolyte application in gel-based EEG systems results in multiple drawbacks, such as long preparation and cleaning time [16], the need to train operators to perform EEG acquisitions [17], inapplicability for long-term acquisition due to gel dehydration and skin irritation effects [17,18], and the risk of gel bridges between neighboring electrodes causing acquisition errors [19]. The aforementioned drawbacks become even more eminent with increasing channel numbers and electrode density [20].

Therefore, dry and semi-dry electrodes have been proposed to address and solve the shortcomings of gel-based systems, enabling fast application, self-application, and mobile use of EEG, while significantly reducing preparation and cleaning efforts. Previous studies reported a reduction in preparation time by about 66% (64-channel dry electrode cap vs. 64-channel gel-based cap) [11], 89% (97-channel dry electrode cap vs. 128-channel gel-based cap) [21], and 69% (256-channel dry electrode cap vs. 256-channel gel-based cap) [22]. The performance of gel-based and dry EEG solutions has been compared by either sequential or simultaneous acquisition with both systems within application-specific paradigms. However, most comparative studies thus far comprise low numbers of volunteers and have been conducted in optimally controlled environments, such as shielded acquisition chambers [23] or focused on low-density EEG with dedicated electrode layouts and applications [3,4,5,24,25,26], including manual electrode placement and preparation [27,28]. Moreover, an important limitation of the majority of previous studies is the single-center and single-operator approach. In contrast to single-center and specifically single-operator studies, multi-center studies provide important—otherwise inaccessible—feedback on the reproducibility and generalizability of findings to different user populations, different environmental conditions, and recording setups. Supporting the aim to increase the accessibility of EEG by simplifying its application, multi-operator studies also provide crucial additional information on the interrelation of operator experience, preparation time, and complexity as well as wearing comfort on the EEG quality. This information contributes to objectively evaluating the existing system performance and identifying key aspects for further improvement both on the technological and operator training level.

We conducted a multi-center, multi-operator study comparing commercial gel-based and dry EEG systems with 64 electrodes. We aim to investigate the reproducibility of results from previous single-center studies while focusing on examining the influence of preparation time, operator experience, increased range of head shapes and hairstyles as well as eventual comfort differences on the performance of gel-based and dry electrode caps.

## 2. Materials and Methods

### 2.1. Experimental Overview

**Figure 1:**
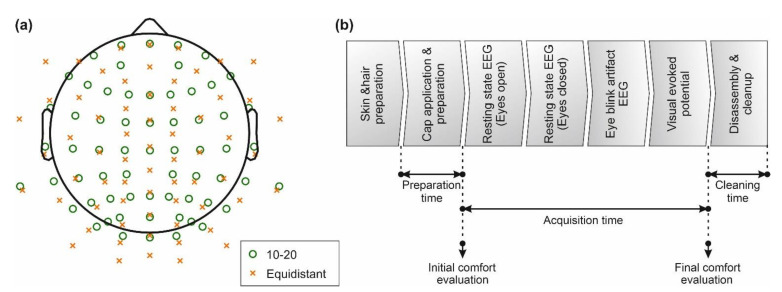
EEG layout and paradigm overview: (**a**) the two electrode layouts used for the EEG data acquisition. Green circles indicate positions according to the extended ten-twenty layout, whereas orange crosses indicate positions of the equidistant layout; (**b**) measurement paradigm and an indication of preparation time, acquisition time, cleanup time, as well as time points of comfort evaluation.

Each participating EEG center compared both commercial dry and gel-based EEG caps with 64 channels. For this purpose, a common experimental protocol was established according to Figure 1b, to assess cap performance for standard clinical EEG in the frequency range of 1–40 Hz. The common protocol was executed identically by all operators at all EEG centers. Moreover, all EEG centers used acquisition and stimulation setups as similar as possible, as described in the following paragraphs.

All EEG recordings were performed using a referential 64-channel amplifier and proprietary acquisition software (eego EE-225, ANT Neuro B.V., Hengelo, The Netherlands). This DC-EEG amplifier provides a 24-bit resolution. Both caps were used with the amplifier’s referential EEG channel dynamic range setting of 750 mV. The referential input noise is < 1.0 µV_rms_ according to manufacturer specifications.

Dry EEG data were recorded with silver/silver chloride (Ag/AgCl)-coated polyurethane (PU) multipin electrodes (waveguard touch, ANT Neuro B.V.) [11,21,22]. The ground and reference electrodes were placed at M1 and M2 positions, respectively, using self-adhesive hydrogel electrodes (Kendall ECG electrodes H124SG, Covidien LLC, Mansfield, United States). No skin preparation was performed at the electrode positions other than skin cleaning at reference and ground using cotton pads soaked with 70% ethanol.

Gel-based EEG data were recorded using EEG caps comprising sintered Ag/AgCl electrodes (waveguard original, ANT Neuro B.V.) in combination with electrolyte gel (Electro-Gel, Electro-Cap International Inc., Eaton, USA). For gel-based recordings with an extended ten-twenty electrode layout, the ground electrodes and reference were placed at AFz and CPz positions, respectively. For gel-based recordings with the equidistant electrode layout, the ground and reference were, respectively, placed at M1 and M2 positions. After cap placement, the hair under each electrode was separated and electrolyte gel injected using a syringe.

The ten-twenty electrode layout [29] and its extensions are the international standard for clinical EEG acquisition, while equidistant electrode layouts are preferred for studies involving spatial filtering [30], source reconstruction, and connectivity analysis [20]. Moreover, the equidistant electrode layout reduces polar average reference (PAR) effects [31,32] and is preferred in dry electrode caps due to more homogeneous mechanical characteristics and adduction [11,21,22,33]. In the study at hand, we included both cap types with ten-twenty and equidistant layouts across sites. With respect to the used comparison metrics, this approach is a worst-case scenario and allows assessment of differences in preparation time, comfort, electrode-skin impedance, and channel reliability.

All acquisitions were carried out in standard EEG laboratories with EEG acquisition and stimulation setups according to ISCEV standards [34]. An overview of all relevant dataset parameters, including electrode layouts, number of volunteers, and operator experience is provided in Table 1. All operators performed an initial training phase including 2 test applications per cap type prior to their participation in the study, in order to ensure sufficient first-hand experience in the correct use of each system.

An overall number of 115 healthy volunteers (82 male and 33 female, average age: 26.7 ± 7.5) participated in the multi-center study. Age range and head circumference between centers have been matched to minimize related influences. The hair length was estimated (44× short (<5 cm), 49× medium (5–15 cm), and 22× long (>15 cm)). The average head circumference was 56.2 ± 5.8 cm. Consequently, the head circumference of all volunteers allowed the use of medium-size caps. The volunteers confirmed not to have a history of drug abuse, no ongoing medical treatment influencing the nervous system, no neurological or skin pathologies, and not being pregnant at the time of participation. For each volunteer, written informed consent was acquired prior to their participation in the study.

The study was executed in accordance with all relevant guidelines, regulations, and ethical standards outlined in the Declaration of Helsinki, and the acquisitions were approved by the respective local Ethics commissions for each participating center.

### 2.2. Acquisition Setup and Paradigm

The acquisitions with the two caps were performed in a randomized sequence with at least 2 h between consecutive cap applications allowing skin recovery.

The preparation time was measured from the time point when the cap was initially applied to the volunteer’s head to when it was ready for EEG recordings. During the preparation of the dry caps, the operator had to ensure all the electrodes have proper contact. For the gel-based caps, EEG gel was injected into each electrode cavity with the requirement of 90% of the electrodes to exhibit impedances < 30 kOhm. Preparation time was recorded for all datasets except dataset 1.

The acquisition time corresponds to the phase between the beginning of the first EEG recording and the end of all EEG recordings. Attention levels based on the Stanford sleepiness scale (scale 1–8, 1 being wide awake) [35] and comfort levels based on Scott and Huskisson pain scale (scale 1–10, 1 being no pain) [36] were documented at the beginning and end of the acquisition phase.

The paradigm of the EEG acquisitions for each volunteer and cap type included recording segments of resting state EEG with eyes open (average duration: 184.0 s), resting state EEG with eyes closed (average duration: 183.0 s), EEG with eye blink artifacts (average duration: 67.1 s), and a pattern reversal visual evoked potential (VEP, 150 epochs, average duration: 204.8 s). Eye blink artifacts were externally triggered: the volunteers had to blink their eyes in response to a beeping sound with a 2 s interval.

Prior to and after each EEG segment, electrode-skin impedances for all electrodes were recorded using the integrated impedance test function of the EEG amplifier.

### 2.3. EEG Data Preprocessing and Analysis

All data processing and analysis were performed in Matlab (R2022a, The Mathworks, Natick, MA, USA). The raw EEG data were exported from the recording software and segmented to separate the individual tests of the paradigm.

Each EEG data segment was filtered using a Butterworth band pass filter with cut-off frequencies of 1 and 40 Hz (24 dB slope), and a 50 Hz notch filter (36 dB slope). Noisy (highly contaminated by artifacts) and isoelectric channels were excluded following a manual inspection. The data at the respective bad channels were interpolated using spherical splines [37]. For acquisitions performed with the ten-twenty layout, data were interpolated to equidistant positions and vice-versa, resulting in 128 channel datasets for each segment. Finally, the interpolated EEG data were re-referenced to common average reference [38]. The combined electrode layouts comprising 128 positions of both the extended ten-twenty and the equidistant layout are depicted in Figure 1a. The data interpolation enables direct comparison of signal characteristics and topographies of acquisitions of the different datasets.

Two segments of resting state EEG were compared: with eyes closed and eyes open. Analysis windows of 30 s duration were extracted from the center of the respective segment and the Welch’s power spectral density (PSD) was estimated [39]. Alpha mean power was then calculated by averaging the power in the frequency band (8–13 Hz).

Eye blink artifacts were segmented into trials of 2 s and 10 s, extracted from the center of the respective stimulation paradigm. The extracted intervals correspond to 1 and 5 triggered eye blinks and are similar to previous studies [21,40,41,42]. The final overlays were obtained by averaging the eye blink artifacts across trials.

VEP data were segmented into 500 ms epochs with a pre-stimulus interval of 100 ms and a post-stimulus interval of 400 ms. Artifactual trials were manually identified and removed across all channels. Subsequently, the remaining trials were averaged. The global field power in the time domain (GFPt) was calculated, corresponding to the spatial standard deviation across all electrodes [43,44]. To compensate for possible variable stimulation-trigger latencies at the different EEG centers, we corrected the latency for each center using the average N75 component peak latency as a reference. For this purpose, we used the acquisition setup of dataset 4 where the latency between the trigger and stimulus presentation was determined prior to the study, measuring both the TTL trigger recorded via the corresponding amplifier input and the stimulus presentation recorded in parallel using a photodiode. During data processing, the VEPs trigger timestamps were corrected to match the stimulus presentation. The data of all other datasets were shifted to match the respective average N75 peak latencies of dataset 4.

Comparison metrics of signal characteristics of gel-based and dry recordings were calculated for the processed data of the eye blink artifacts, resting state EEG, and VEP. These metrics include Pearson correlation coefficient and root mean squared deviation (RMSD). The Kolmogorov–Smirnov Tests (KS Tests) were performed to confirm that the results were not normally distributed [45] and the Wilcoxon–Mann–Whitney U tests (U tests) with an alpha value of 0.05 were performed to test the statistical equality of medians [46]. Moreover, the gel-based and dry mean PSD in the alpha band (8–13 Hz) for resting state with closed eyes were tested using the Mann–Whitney U Test. U Tests were also conducted on N75 and P100 amplitudes of the VEP as well as on the latencies of the N75 and P100 components.

Each of the 64 channels in each of the 4 analysis sequences, 2 caps and 115 volunteers were rated by visual inspection. The binary rating as either “good” or “bad” was used to calculate the relative channel reliability. A channel was defined as bad if it was exhibiting either strong artifacts or being isoelectric during at least 50% of the analyzed data sequence. Consequently, the channel reliability was defined as the ratio between the number of “good” data sequences and the total number of analyzed data sequences of the given channel.

Electrode-skin impedances recorded at the start and the end of the acquisition phase were averaged for each electrode position within each cap/electrode type across all volunteers. 

We compared grand averages of channel reliability, electrode-skin impedance, attention level, comfort level, and preparation time for both cap types across all volunteers and datasets. Furthermore, we calculated individual averages for the aforementioned metrics across all volunteers for individual datasets, to allow a cross-dataset/cross-site comparison of the results.

## 3. Results

### 3.1. Preparation Time, Acquisition Time, Attention, and Comfort

The grand average preparation time, acquisition time, as well as attention and comfort levels reported at the start and end of the acquisition phase are listed in Table 2.

No considerable differences have been observed in the acquisition time and attention levels neither between the two cap types nor between the individual datasets.

**Figure 2:**
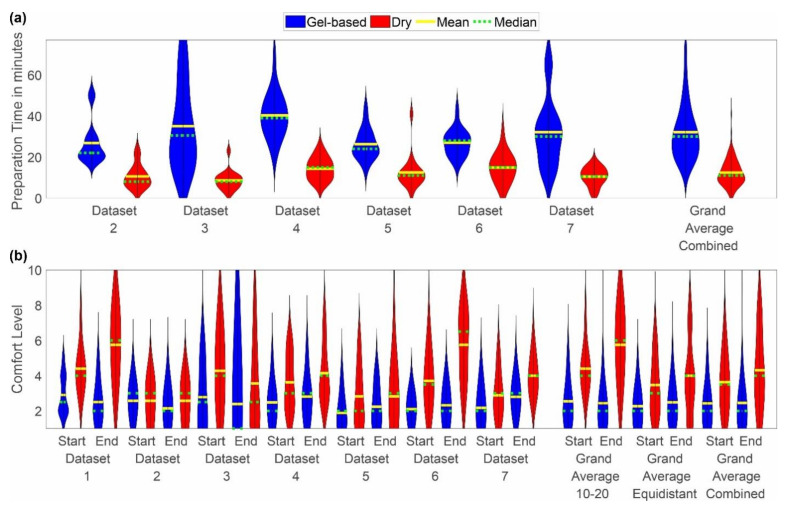
Violin plot of the distributions of (**a**) preparation time and (**b**) comfort level reported as grand average over all datasets, grand average per cap layout, and individual results per dataset. Blue indicates the results for the gel-based caps, whereas red indicates the results for the dry electrode caps. The yellow line indicates the mean, and the green line indicates the median of the distribution. The violin plots have been cut at the minimum and maximum reported values. Note that the preparation time was not recorded for dataset 1.

The distributions recorded for each individual dataset along with the grand average distributions are shown for the two parameters’ preparation time and comfort level in Figure 2a,b respectively.

The mean preparation time of the gel-based caps ranged from 11 min in dataset 3 to 77 min in dataset 3. For all datasets, the mean preparation time of the dry caps was considerably shorter, ranging from 2 min in datasets 3, 5, and 6 to 41 min in dataset 5. No considerable difference was observed between the mean preparation time of the gel-based caps with extended ten-twenty and equidistant layout.

The comfort rating of the caps varied considerably between the individual datasets and for both cap types. For each dataset, the comfort value reported for the gel-based cap is lower than or equal to the value reported for the dry electrode cap, indicating higher comfort with the gel-based caps. Furthermore, for the majority of datasets, the comfort value at the beginning of the acquisition phase is lower (i.e., higher comfort) as compared to the value at the end. This observation holds for both cap types. Exceptions can be noticed for dataset 1 (average gel-based comfort value is lower at the end), dataset 2 (average gel-based comfort value is lower at the end; dry comfort values are equal), and dataset 3 (lower average comfort values are at the end for both cap types). Overall, the highest comfort values (i.e., lowest comfort) were reported for the dry caps in dataset 1 and dataset 6.

### 3.2. Impedance and Reliability

**Figure 3:**
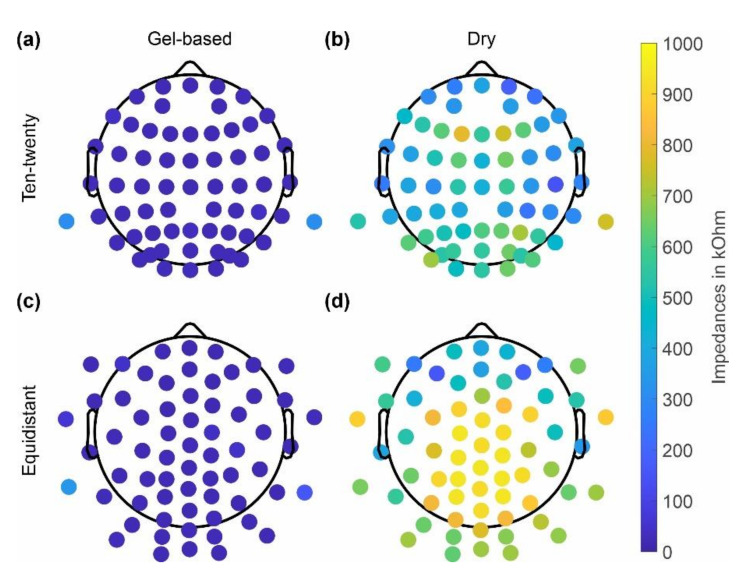
Grand average electrode-skin impedances calculated across all volunteers using the respective cap type: (**a**,**c**) gel-based; (**b**,**d**) dry electrodes; and (**a**,**b**) extended ten-twenty layout; and (**c**,**d**) equidistant layout.

The skin-electrode impedances of the gel-based and dry caps averaged over all volunteers are shown in Figure 3 separately for caps with the ten-twenty layout and the equidistant layout. The dry cap showed higher average impedances of 464 ± 394 kOhm (ten-twenty layout), 685 ± 396 kOhm (equidistant layout) compared to the gel-based cap 20 ± 106 kOhm (ten-twenty layout) and 31 ± 108 kOhm (equidistant layout). Moreover, an increased impedance level at the central and parietal positions is evident for the dry electrode caps with both layouts.

**Figure 4:**
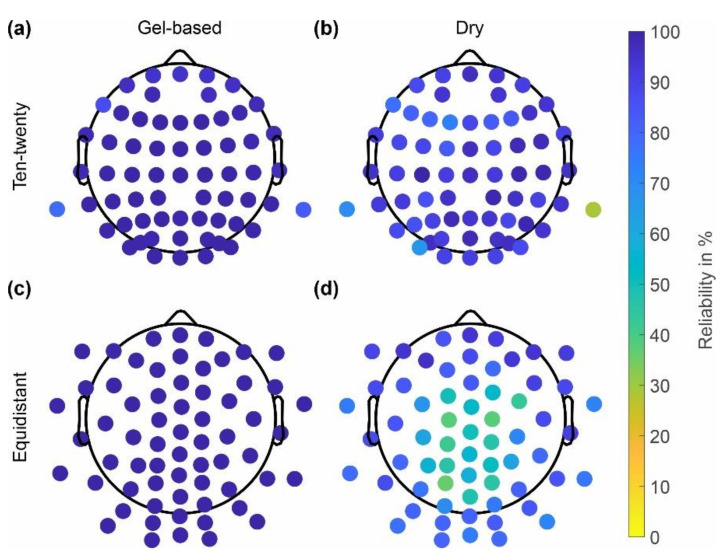
Channel reliability calculated across all recordings performed using the respective cap type: (**a**,**c**) gel-based; (**b**,**d**) dry electrodes; (**a**,**b**) extended ten-twenty layout; and (**c**,**d**) equidistant layout.

Similar to the skin-electrode impedances, the reliability of channels in the gel-based and dry caps with extended ten-twenty and equidistant layouts are shown in Figure 4. The gel-based cap showed higher reliability of 98 ± 3% (ten-twenty layout), 99 ± 0% (equidistant layout) as compared to the dry electrode cap, which showed reliability values of 89 ± 10% (ten-twenty layout) and 72 ± 17% (equidistant layout). Decreasing channel reliability at central and parietal positions is evident for the dry electrode caps with an equidistant layout. The channels with reliability < 70% match the electrode positions with electrode-skin impedances > 800 kOhm.

**Figure 5:**
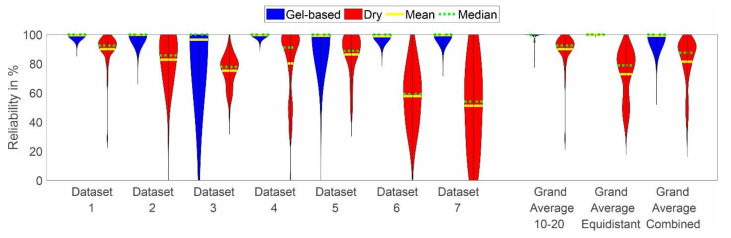
Violin plot of the channel reliability distributions of all datasets, including grand average per cap type, layout, and individual results per dataset. Blue indicates the results for the gel-based caps, while red indicates the results for the dry electrode caps. The yellow line indicates the mean, and the green line indicates the median of the distribution. The violin plots have been cut at the minimum and maximum reported values.

The distribution of the channel reliability across cap types, layouts, and datasets is shown in Figure 5. In line with the observations based on Figure 4, the channel reliability of the dry caps is lower than the reliability of the gel-based caps in all datasets. However, strong variability is evident across datasets, with the lowest mean channel reliabilities of 57% and 49% achieved in datasets 6 and 7, respectively. Moreover, increased variability of the gel-based recordings is visible in datasets 3 and 5 as compared to the other datasets.

### 3.3. EEG Signal Characteristics

**Figure 6:**
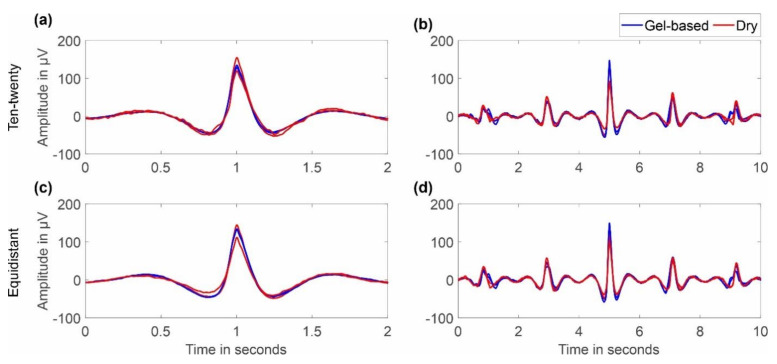
Grand average comparison (over all respective volunteers) of eye blinks recorded with gel-based and dry electrode caps: (**a**,**b**) ten-twenty layout/channels Fp1 and Fp2; (**c**,**d**) equidistant layout/channels 1L and 1R; (**a**,**c**) overlay of 2 s; and (**b**,**d**) overlay of 10 s.

Figure 6 shows an overlay of the grand averages of eye blink artifacts recorded with the two caps and layout types. For the caps with an extended ten-twenty layout, we selected channels Fp1 and Fp2, while for the caps with the equidistant layout, we selected channels 1L and 1R, respectively. Overlays are shown for 2 s and 10 s duration. It can be seen that the shape of the artifact is similar for gel-based and dry caps and for both layouts. No considerable difference in the grand average artifact amplitude or duration can be observed between the electrode types, indicating similar general sensor characteristics.

**Figure 7:**
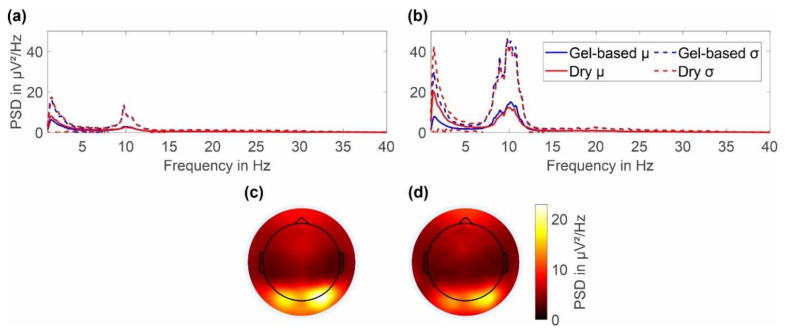
Grand average power spectral density of the gel-based and dry electrode recordings calculated over all volunteers and both cap layouts: (**a**) PSD for resting state EEG with eyes open and (**b**) resting state EEG with eyes closed. Topographic plot of the alpha band power for (**c**) gel-based and (**d**) dry electrodes during resting state with eyes closed.

The average power spectral density of the resting state EEG with eyes open and eyes closed are respectively shown in Figure 7a,b together with the 2D topographic representation of the mean power in the alpha band (8–13 Hz) during eyes closed for the gel-based and dry electrode caps (Figure 7c,d).

In the frequency range of 1 to 40 Hz, the gel-based and dry PSDs show similar spectral signal characteristics for both cap types. Both PSDs strongly overlay each other in both the mean and standard deviation. Alpha band activity is evident for both the recordings with eyes open and eyes closed, with a clearly increased activity for the latter. During eyes closed, the alpha peak power for dry electrode recordings is 12.31 ± 20.39 μV^2^/Hz at 10.01 Hz, whereas for the gel-based recording the alpha peak power is 15.04 ± 30.16 μV^2^/Hz at 10.13 Hz. A remarkable increase in power can be observed in the PSD of the dry cap for frequencies below 6 Hz. However, throughout the investigated frequency range, the differences in mean PSD of both caps are lower than the respective standard deviations.

For the 2D topographic plots of the alpha band power, the color maps were normalized to the maximum value of both gel-based and dry caps. An increased alpha band power can be seen to be concentrated in the lower parietal and occipital regions for both gel-based and dry caps. No considerable differences in the power distributions are evident.

**Figure 8:**
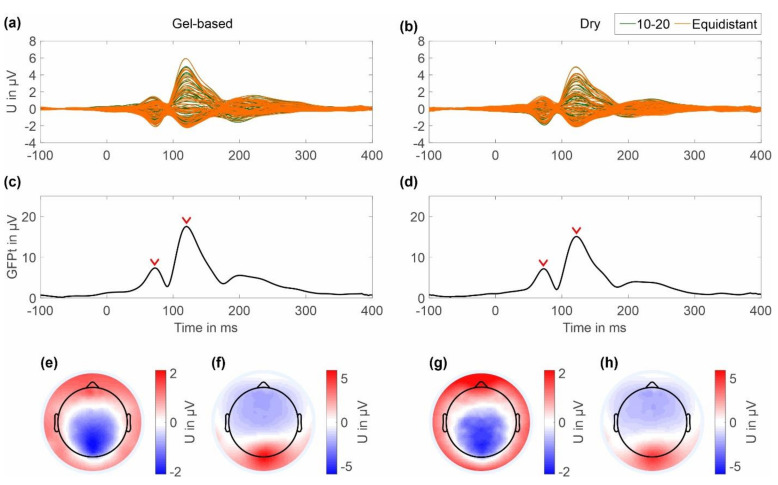
Grand average VEP results of the gel-based cap and dry cap calculated over all volunteers and both layouts: (**a**,**c**,**e**,**f**) gel-based and (**b**,**d**,**g**,**h**) dry electrode results; (**a**,**b**) butterfly plot of all 64 ten-twenty (green) and equidistant (orange) electrodes; (**c**,**d**) GFPt calculated over all channels; red arrows indicate time points of N75 and P100 component peak amplitude (**e**,**g**) 2D interpolated topographic plots of the N75; and (**f**,**h**) P100 components of the VEP at peak amplitude, respectively. Colormap scales of the topographic plots have been scaled to the absolute maximum amplitude of the respective component in both cap types.

The results of the checkerboard pattern reversal VEP averaged across all clean trials for a period of 100 ms pre-stimulus and 400 ms post-stimulus are shown in Figure 8. The butterfly plots in Figure 8a,b show the grand average for all channels for the gel-based and dry cap, respectively. The resulting GFPts are plotted for both caps in Figure 8c,d respectively. Two post-stimulus signal peaks can be seen representing the N75 and P100 components of the VEP. The peak GFPt of the N75 component is 7.38 μV at 72 ms for the gel-based cap and 7.18 μV at 72 ms for the dry electrode cap, respectively. The peak GFPt of the P100 component is 17.57 μV at 120 ms for the gel-based cap and 15.11 μV at 122 ms for the dry electrode cap, respectively.

In the 2D topographic plots in Figure 8e,f the color maps were normalized to the maximum value for both the gel-based and dry caps. The topographic maps show the expected electric potential distribution and orientation for the N75 components and the P100 components for the gel-based and dry caps. No considerable differences in the electric potential distributions of the two cap types can be observed.

When the grand averages were considered, the correlation between the gel-based and dry electrode signals was > 0.9 for all metrics. The statistical results for correlation and RMSD calculated over all subjects for the different EEG segments are listed in Table 3. For resting state EEG, we compared the PSD of the recordings with eyes closed. For eye blink artifacts, we compared the averaged trials per channel Fp1/Fp2 (extended ten-twenty layout) and L1/R1 (equidistant layout) in the time domain for a window of 2 s. For the VEP, we compared a) averaged trials per channel and b) GFPt. No statistically significant differences were found for the PSD of spontaneous EEG with eyes closed (*p* ≥ 0.14) nor for amplitudes or latencies of the VEP (*p* ≥ 0.31).

All the analyzed metrics passed the KS Test, indicating that they are not normally distributed. For all metrics, the U Tests resulted in *p* values higher than the set alpha value of 0.05. The statistical results, therefore, suggest that there are no statistically significant differences between the signals acquired using gel-based and dry electrode caps.

## 4. Discussion

We performed a multi-center study across several countries comparing commercial 64-channel gel-based and dry electrode caps for EEG acquisition. The performance of the two systems was compared in terms of preparation and acquisition time, attention and comfort level, electrode-skin impedance, channel reliability, and EEG signal characteristics using an established paradigm. Our findings are in line with previous publications and validation studies of multipin-shaped dry electrodes. Beyond reproducing findings of previous publications in a multi-center and multi-operator setup and a larger group of volunteers, the study at hand provides evidence for the relationship between preparation time, comfort, and operator experience with the channel reliability of the dry electrode systems. Consequently, our study contributes to further validation of the multipin dry electrodes and provides evidence for the reproducibility and generalizability of the performance of these dry electrodes in different environments and for different operators.

### 4.1. Preparation and Acquisition Time, Attention, and Comfort

The average preparation time for the dry cap is 38% of the average preparation time of the gel-based cap. This significant reduction in preparation time is in line with previous publications [11,21,22] and was most likely to be ascribed to the fact that dry cap systems do not need time-consuming skin preparation and gel application. 

No considerable difference was observed between the gel-based and the dry cap for the acquisition time and the reported attention level before and after acquisition. This may be caused by the fact that, although the datasets have been acquired at different sites, the acquisition paradigm was identical across sites. It is worth noting that all volunteers, after the acquisition with the gel-based caps, had to spend additional time to clean their hair from gel residuals. Similarly, operators also had to spend more time cleaning the gel-based caps.

Volunteers generally expressed that gel-based caps were slightly more comfortable than the dry electrode caps, primarily because of a needle-like sensation caused by the dry electrodes contacting the scalp and of individual spots of (subjectively) increased contact pressure. Again, this observation is in line with previous publications [11,21]. The multi-center results seem to confirm this fact for different populations, including a wider range of head shapes and hairstyles. Consequently, a softer and more adaptive electrode shape that can maintain enough adduction force to the scalp while avoiding excessive pressure will improve the comfort of future dry electrode generations. Further improvement of comfort may be achieved by a more flexible cap or headset designs capable of increased adaptivity to different head shapes. 

The preparation time, acquisition time, attention level, and comfort level remained without considerable differences between caps with equidistant or ten-twenty layouts.

### 4.2. Impedance and Reliability

As seen in Figure 2 and Figure 3, the skin-electrode impedance for the dry caps was higher across the majority of electrodes. The impedances were increased particularly in the central and parietal region of the head. No considerable differences between the two layouts were evident. In comparison with the results of previous studies [11,21,22], the higher skin-electrode impedance for the dry caps could be seen as a general limitation of this type of textile cap, which would require an adjusted concept and design to improve adduction at these positions while maintaining adduction level at circumferential head positions. 

Even though the dry EEG caps (of both layouts) had in general higher impedance levels, those did not necessarily result in bad channels. Only the positions with very high impedance levels seem to correspond to the channels of lower reliability, in line with previous findings [22]. However, individual channels with high impedances at circumferential electrode positions do provide good signals. Consequently, the decreased reliability of some channels is unlikely related to the high impedance values but to an insufficient adduction, which may result in instable electrode-skin contacts. It is important to note that the dry electrode cap with the ten-twenty layout was used only in dataset 1. Therefore, those results cannot be generalized and need to be further investigated.

The results do not indicate a direct relationship between (a) preparation time and channel reliability, nor (b) comfort and channel reliability. Consequently, spending additional time for dry electrode preparation or compromising on comfort might not contribute to better electrode performance. In fact, excessive electrode pressure and improper application may contribute to a decrease in the wearing comfort and an increase in the risk of mechanical damage to electrodes and caps. They may also lead to increased artifacts related to skin compression, skin stretching, and sweating [47,48].

Operator experience seems to play an important role in the cap performance, given that the datasets with the lowest channel reliability correspond to the operators with the least experience with both gel-based and dry EEG. Moreover, prior experience with gel-based EEG systems does not directly translate to improved results in dry electrode applications. This fact underlines the importance of training and knowledge about the specificities of dry electrode application. Dedicated operator training is required for optimal results, including the following considerations:

- Selection of the correct EEG cap size considering multiple factors, such as subject head circumference, head shape, hairstyle, and hair density;
- Clean skin and hair, avoiding fat and oil layers, which would increase electrode-skin impedance and decrease electrical contact quality;
- Stable and low electrode-skin impedances at the ground and reference electrodes influencing the signal quality of all referential channels;
- Correct application procedure, including minimal post-application movements of the cap on the head, to avoid hair accumulation and counter-pressure at individual electrode areas;
- Dry-electrode specific knowledge on identification and improvement of bad or instable electrode-skin contacts.

### 4.3. EEG Signal Characteristics

No significant difference could be identified between the gel-based and dry cap for resting state EEG, eye blink artifacts, and VEPs. The results are within inter-trial and intra-individual variability margins [21]. Overall and in line with previous studies, after the exclusion of bad channels, the dry electrodes show equivalent signal quality in all comparison metrics [11,21,22]. Moreover, no differences in the characteristics of EEG signals acquired with caps with equidistant or extended ten-twenty layouts were observed. The applied EEG paradigm and signal metrics cover a broad range of signal characteristics allowing a comprehensive assessment of cap and electrode performance in line with metrics used in related previous studies [11,21,22,23,24,25,26,27,28].

The increased low-frequency power of the dry electrodes for resting state EEG with eyes closed may be related to unnoticed eye movements or skin effects. This phenomenon was already reported in previous studies [19,21]. However, the differences in the mean PSD were below the respective standard deviation of both electrode types.

No difference in the N75 component latency and only a small difference of 2 ms in latency of the P100 components were observed. This low difference in latency was most likely due to intra-individual variability. 

The main difference observed between the gel-based and dry electrode caps is the higher impedance and lower channel reliability, especially at the central and parietal regions, while considerably reducing the cap preparation time and subsequent cleaning effort. This motivates future work on a cap design that can provide homogenous adduction across the whole head. In addition, the material and shape of electrodes could be further improved to provide maximum comfort and adaptivity to different head shapes and to cope with different kinds of hair. Adapted electrode and cap designs may also be developed for other applications, such as sleep EEG, mobile EEG, and neonatal EEG. 

## 5. Conclusions

In conclusion, we conducted a multi-center evaluation of gel-based and dry caps across several countries. The results of our study confirm the findings of previous studies in terms of dry electrode signal quality and reliability and provide evidence for the reproducibility and generalizability of the performance of these dry electrodes. Most important, the results pinpoint the importance of dry-electrode-specific operator training and experience for optimal performance. On the contrary, extensive preparation times and compromised comfort (including excessive pressure) may even compromise dry electrode performance. Future improved cap and electrode designs, as well as dry-electrode-specific operator training, may support increased use of this promising technology in a wide range of new applications, such as mobile EEG, telemedical EEG, personal, and home use.

## Figures and Tables

**Table 1:** Overview of the multi-center study datasets including volunteer information (age and head circumference represent mean ± standard deviation), electrode layout, and operator experience.

Dataset	EEG Center	Volunteer No.	Age (Years)	Head Circumference (cm)	Electrode Layout		Operator Experience (Years)	
					Gel- Based	Dry	Gel- Based	Dry
1	Swinburne University of Technology, Australia	20	33.4 (±10.4)	56.8 (±1.9)	ten-twenty	ten-twenty	>5	<1
2	University ‘G. d’Annunzio’ of Chieti–Pescara, Italy	7	28.0 (±5.4)	56.9 (±1.9)	equidistant	equidistant	<1	<1
3	University ‘G. d’Annunzio’ of Chieti–Pescara, Italy	18	27.1 (±5.9)	56.6 (±1.2)	ten-twenty	equidistant	>5	>1
4	Technische Universität Ilmenau, Germany	21	26.3 (±3.7)	56.5 (±1.4)	equidistant	equidistant	>5	>5
5	Universidade do Porto, Porto, Portugal	17	26.3 (±8.7)	55.9 (±1.4)	equidistant	equidistant	>1	>1
6	Chinese University of Hong Kong, China	22	22.2 (±3.0)	56.2 (±2.8)	ten-twenty	equidistant	<1	<1
7	Universiti Teknologi Malaysia, Malaysia	10	21.2 (±0.9)	55.8 (±0.8)	ten-twenty	equidistant	<1	<1

**Table 2:** Grand average preparation time, acquisition time, attention levels, and comfort level calculated over all datasets.

Cap Type	Preparation Time (min)	Acquisition Time (min)	Attention Level (1–10)		Comfort Level (1–10)	
			Start	End	Start	End
Gel-based	32.3 ± 13.8	21.3 ± 9.3	3.3 ± 1.6	3.6 ± 1.9	2.4 ± 1.3	2.5 ± 1.4
Dry	12.4 ± 6.5	23.4 ± 8.3	3.2 ± 1.6	3.7 ± 1.9	3.6 ± 1.8	4.3 ± 2.2

**Table 3:** Comparison metrics and statistical test results for the signal characteristics of the EEG acquired using gel-based and dry electrode caps: correlation, RMSD.

EEG Segment	Electrode Layout	Average Correlation	Average RMSD
Eye blink artifact (Fp1/Fp2 and L1/R1)	ten-twenty	0.83 ± 0.13	36.45 ± 32.51 μV
	equidistant	0.87 ± 0.11	27.24 ± 23.25 μV
Resting state closed eyes (PSD, all channels)	combined layout	0.70 ± 0.16	2.14 ± 3.93 μV^2^/Hz
VEP (all channels)	combined layout	0.66 ± 0.28	0.82 ± 0.44 μV
VEP (GFPt)	combined layout	0.85 ± 0.17	2.97 ± 1.53 μV

## Data Availability

Raw data and analysis scripts are available upon request to the corresponding author.
